# Acute exposure to nocturnal train noise induces endothelial dysfunction and pro-thromboinflammatory changes of the plasma proteome in healthy subjects

**DOI:** 10.1007/s00395-019-0753-y

**Published:** 2019-10-29

**Authors:** Johannes Herzog, Frank P. Schmidt, Omar Hahad, Seyed Hamidreza Mahmoudpour, Alina K. Mangold, Pascal Garcia Andreo, Jürgen Prochaska, Thomas Koeck, Philipp S. Wild, Mette Sørensen, Andreas Daiber, Thomas Münzel

**Affiliations:** 1 Cardiology I, Center for Cardiology, University Medical Center of the Johannes Gutenberg-University Mainz, Langenbeckstr. 1, 55131 Mainz, Germany; 2 Department of Biometry and Bioinformatics, Institute for Medical Biostatistics, Epidemiology, and Informatics (IMBEI), University Medical Center of the Johannes Gutenberg University, Mainz, Germany; 3 Center for Thrombosis and Hemostasis, University Medical Center of the Johannes Gutenberg University Mainz, Mainz, Germany; 4 Preventive Cardiology and Preventive Medicine, Center for Cardiology, University Medical Center of the Johannes Gutenberg-University Mainz, Mainz, Germany; 5 German Center for Cardiovascular Research (DZHK), Partner Site Rhine-Main, Mainz, Germany; 6 Danish Cancer Society, Copenhagen, Denmark; 7 Department of Natural Science and Environment, Roskilde University, Roskilde, Denmark; 8 Klinikum Mutterhaus der Borromäerinnen gGmbH, Trier, Germany

**Keywords:** Environmental risk factor, Train noise exposure, Flow-mediated dilation, Oxidative stress, Systemic inflammation, Pro-thrombotic state, Sleep deprivation

## Abstract

Nocturnal train noise exposure has been associated with hypertension and myocardial infarction. It remains unclear whether acute nighttime train exposure may induce subclinical atherosclerosis, such as endothelial dysfunction and other functional and/or biochemical changes. Thus, we aimed to expose healthy subjects to nocturnal train noise and to assess endothelial function, changes in plasma protein levels and clinical parameters. In a randomized crossover study, we exposed 70 healthy volunteers to either background or two different simulated train noise scenarios in their homes during three nights. After each night, participants visited the study center for measurement of vascular function and assessment of other biomedical and biochemical parameters. The three nighttime noise scenarios were exposure to either background noise (control), 30 or 60 train noise events (Noise30 or Noise60), with average sound pressure levels of 33, 52 and 54 dB(A), respectively. Flow-mediated dilation (FMD) of the brachial artery was 11.23 ± 4.68% for control, compared to 8.71 ± 3.83% for Noise30 and 8.47 ± 3.73% for Noise60 (*p *< 0.001 vs. control). Sleep quality was impaired after both Noise30 and Noise60 nights (*p *< 0.001 vs. control). Targeted proteomic analysis showed substantial changes of plasma proteins after the Noise60 night, mainly centered on redox, pro-thrombotic and proinflammatory pathways. Exposure to simulated nocturnal train noise impaired endothelial function. The proteomic changes point toward a proinflammatory and pro-thrombotic phenotype in response to nocturnal train noise and provide a molecular basis to explain the increased cardiovascular risk observed in epidemiological noise studies.

**Electronic supplementary material:**

The online version of this article (10.1007/s00395-019-0753-y) contains supplementary material, which is available to authorized users.

## Introduction

The Lancet Commission on pollution and health and the Global Burden of Disease (GBD) study estimate that all forms of pollution caused 9–12.6 million deaths in 2012 and 2015, respectively, reflecting 16–20% of total mortality worldwide [24]. These values are most likely underestimated as recent global environmental mortality models found more than 8 million premature global deaths in 2015 due to air pollution by particulate matter with a diameter ≤ 2.5 µm (PM_2.5_) alone [8, 25]. Most importantly, these reports focused on chemical pollution but neglected the contribution of non-chemical risk factors, such as climate parameters and noise exposure.

Environmental noise and air pollution coexists in urban environments. While air pollution is already an established cardiovascular risk factor [10], noise is increasingly acknowledged as risk factor for various major diseases and conditions [36] (for reviews, see [30, 33]). Although a large proportion of the population is exposed to noise levels exceeding the guidelines values combined with growing evidence linking traffic noise to cardiovascular morbidity and mortality, traffic noise is neither mentioned as a health risk factor in the GBD Study (only occupational noise is mentioned) [11] nor in the report “Health at a Glance: Europe 2018” [37]. Strong epidemiological evidence was provided by the WHO environmental noise guidelines for the European region, concluding that exposure to traffic noise increases risk for non-auditory diseases (not associated with hearing loss), mainly by cardiovascular development/progression, and potentially metabolic disease [22]. According to the WHO guidelines, the pooled relative risk for ischemic heart disease was 1.08 (95% CI 1.01–1.15) per 10 dB(A) increase in traffic noise exposure, starting at 53 dB [22].

Exposure to railway and road traffic noise has been associated with an increase in arterial stiffness, a subclinical marker of atherosclerosis and development of future cardiovascular disease (CVD) [13]. Although the strength of the associations varies significantly across published studies, chronic exposure to road, railway or aircraft noise seems to be associated with elevated blood pressure, arterial hypertension, stroke, increased use of antihypertensive medication, increased incidence of heart failure, atrial fibrillation and arrhythmia [15, 30, 33].

According to the WHO, at least 1 million healthy life years are lost annually from traffic-related noise in Western Europe [53]. The annual noise-related CVD burden is substantial in Europe, as an estimation found environmental noise to result in 1.7 million cases of hypertension, 80,000 hospital admissions, and at least 18,000 excess deaths [19]. It was estimated that reducing noise levels by 5 dB(A) could reduce hypertension by 1.4% and ischemic heart disease by 1.8%, saving 3.9 billion dollars in health costs [54]. The WHO, however, indicates the urgent need for additional evidence, based on both longitudinal studies and experimental studies, to assess the cardiovascular and metabolic adverse health impacts of noise [22].

In field studies, we recently demonstrated that acute simulated nighttime aircraft noise can induce endothelial dysfunction reflecting subclinical atherosclerosis, can increase stress hormone release, worsen sleep quality and can cause an increase in blood pressure [45, 46]. Also acute noise exposure during the daytime under laboratory conditions caused impaired autonomous function in normotensive and hypertensive subjects characterized by increases in systolic/diastolic blood pressure, heart rate, and muscle tone [48]. According to the noise reaction model introduced by Babisch, the so-called “indirect pathway” plays a crucial role in causing CVD [4]. It represents cognitive perception of noise, subsequent cortical activation, leading to increased levels of stress hormones that become manifest in CVD, including acute myocardial infarction, heart failure, hypertension, arrhythmia and stroke [5, 33], but potentially also TAKOTSUBO-syndrome [32] and other stress-triggered CVD [12]. It is proposed that a perturbation of the autonomic nervous system, and/or sympathoadrenal activation [42], and increase in cortisone levels, [50] the release of proinflammatory mediators, modified lipids or phospholipids and activation of leukocyte populations, endothelial dysfunction and activation of pro-thrombotic pathways are crucial [30, 33]. Recent animal studies have found an essential role of oxidative stress, impairment of the circadian clock and dysregulation of gene networks leading to endothelial dysfunction, and vascular/cerebral damage from aircraft noise in particular when the animals were exposed to noise during the sleep phase reflecting nighttime noise [23].

With the present study, we sought to determine the effects of train noise exposure on vascular function, which has not been investigated previously. With the opening of the Gotthard rail tunnel linking Switzerland and Italy, longer freight trains are expected to operate on the vital Rhine-Alpine rail freight corridor by 2021, thus further increasing the noise burden along this corridor. To address this, we conducted a field study investigating the impact of different train noise scenarios during nighttime on endothelial function, an established prognostic marker for future cardiovascular events [31]. We also investigated the involvement of oxidative stress in effects of noise on endothelial dysfunction, by treating a subgroup of noise-exposed subjects with the antioxidant vitamin C [31]. In addition, we performed targeted immuno-PCR-based proteomic analysis in plasma to gain new mechanistic insights into noise-driven pathophysiological changes.

## Materials and methods

### Study population and ethical aspects

All human data were collected in accordance with the declaration of Helsinki and ethical approval was granted by the Landesärztekammer Rheinland-Pfalz [Mainz, Germany; permit number: 837.265.16 (10584)]. Written consent was received from all included individuals. We excluded anti-traffic noise activists and persons with high nighttime traffic noise exposure at home, determined by noise maps available from municipal online resources [A-weighted equivalent continuous sound level (*L*_Aeq_), 22–6 h, 45 dB(A) for rail traffic, road traffic and aircraft noise]. Also, persons with sleeping disorders were indicated by a score > 10 on the Pittsburgh Sleep Quality Index (PSQI) [59] or psychiatric disorders assessed by M.I.NI. Screen interview [52] was excluded. An age-adjusted hearing loss of 30 dB(A) or more, indications for obstructive sleep apnea in the screening test, current shift work or regular drug intake except oral contraceptives led to an exclusion from the study. The study enrolled 70 healthy non-smokers between 18 and 60 years old. In female participants, care was taken to synchronize study nights with the hormonal status.

### Study procedures

We conducted a blinded study of nighttime train noise exposure in healthy volunteers. After inclusion, participants underwent three study nights and in the morning after each study night, they went to the study center (all measurements were performed before 10 a.m.). There was an exposition to one of the three noise scenarios in each study night in a randomised manner. Noise scenarios were labeled as control (C), Noise30 and Noise60: The control scenario contained no “playback-generated” noise events, but the subjects were exposed to normal background noise present in their home environments (peak sound level 65 dB(A)). Noise30 and Noise60 consisted of playback of train noise events with 30 and 60 noise events, respectively, each event with a peak sound level of 73–75 dB(A) as described below. The sequence of noise and control nights for each participant was determined according to the randomisation plan with six different sequences possible: C–Noise30–Noise60, C–Noise60–Noise30, Noise30–C–Noise60, Noise30–Noise60–C, Noise60–C–Noise30, Noise60–Noise30–C, resulting in investigator and participant blinding for the noise scenario sequence at study onset. Study nights were prescheduled to ensure a minimum of three non-study nights between study nights and if possible, on same weekday. In premenopausal women, care was taken to schedule study nights in the same phase of the hormone cycle. Intake of caffeine containing beverages, alcohol or supplemental vitamins was not allowed the day before, during and in the morning after each study night. Apart from that, participants were advised to stick to their normal routine, especially with regard to their usual sleep–wake rhythm. Study nights took place in the familiar surroundings of the participants’ own bedrooms, with the goal of minimizing effects of an artificial laboratory situation.

The two train noise scenarios contained four different train noise events, each caused by a passing train. These events were recorded under controlled circumstances in a bedroom of a resident living near an important railway track of Germany located in the Mittelrheintal (Kamp-Bornhofen, near Boppard/Koblenz) being part of the Rhine-Alpine rail freight corridor Rotterdam-Genoa. Recordings took place between 10 p.m. and 6 a.m. with window tilted open and microphone placed 0.15 m above the headboard in an actual bedroom. The recordings were conducted by a specialized independent engineering office (Schalltechnisches Ingenieurbüro Pies GbR, Birkenstraße 34, 56154 Boppard, Germany). Noise patterns were played back as MP3 files via customary portable audio systems, which were positioned 1 m above the floor at the end of the bed. To ensure compliance, sound pressure level (SPL) was continuously measured via class-2 sound level meters (Extech Datenlogger 407780A, 30–130 dB, Extech Datenlogger 407764, 3–130 dB.), which were placed near to the head of the participant.

The train noise scenarios started with playback of a 30 s lasting tone signaling the beginning of the study night and enabling checking of the equipment. This was followed by 45 min of silence to enable subjects to fall asleep, after which the first noise event was played. Four different noise events, each representing a different train passing by, were repeatedly played back and lasted for 61 (train 1 and 4), 71 (train 2) and 77 (train 3) seconds, respectively. Maximum sound pressure level was 74.9 dB(A) for train 1, 73.1 for train 2, 73.8 for train 3 and 74.6 for train 4. Noise scenarios started with train 1 followed by train 2, 3 and 4; afterwards, the sequence was starting again. For the Noise30 scenario, the sequences of the four trains were repeated 7.5 times ending with train 2, and for the Noise60 scenario it was repeated 15 times, ending with train 4. Time between noise events followed a long–short–long pattern (time between events in Noise30 approximately 15.3 min or 7.7 min, respectively, and in Noise60 approximately 6.8 min or 3.4 min, respectively). The last event was played back after roughly 416 min (suppl. Figure S1).

### Functional, biochemical and clinical chemistry parameters

During study nights, oxygen saturation (SpO_2_), electrocardiogram and derived parameters as described in previous studies (blood pressure, Puls Transit Time, heart rate acceleration) [6, 14, 39] were continuously measured by wearing portable polygraphic screening devices (SOMNO Watch™plus or SOMNO touch™, SOMNOmedics GmbH, Randersacker, Germany).

After each study night, participants came to the study center. All measurements were conducted and all samples collected before 10 a.m. Fasting state was obligatory. Flow-mediated dilatation (FMD) of the brachial artery was measured using standardized methods [34, 38, 47]. To determine the effect of reactive oxygen species, 30 out of the 70 participants were randomly chosen and orally administered 2 g of vitamin C directly after initial measurement of FMD, which was followed 2 h later by a second FMD measurement (on the same day as the initial FMD measurement without vitamin C) using an exactly similar protocol for vitamin C administration as previously published [41]. This original study reported plasma levels of vitamin C of 42 ± 21 mM (prior) versus 120 ± 54 mM (post). A placebo group was not included in our study design since previously placebo showed no effect versus vitamin C in a crossover design [41]. Vitamin C administration was previously shown to allow measurement of the impact of oxidative stress burden on endothelial function (FMD) [17].

Afterwards, blood samples were drawn and immediately analyzed by our in-house clinical chemistry laboratory. An aliquot of the samples was centrifuged and stored at − 80 °C for further testing.

For measurement of global noise sensitivity, the Dortmund Noise Sensitivity Questionnaire (NoiSeQ) [49] was used. To determine the chronotype of each participant, Horne–Ostberg Morningness-Eveningness Questionnaire (MEQ) [18] was used. A questionnaire consisting out of 19 items was used to assess the participants’ attitude toward train noise with higher values denoting a more negative attitude. Serum levels of catecholamines (dopamine, adrenaline and noradrenaline) and 8-isoprostane were measured by commercial ELISA kits according to the vendors’ protocols.

### Targeted proteomics

To elucidate molecular manifestations of train noise on mechanisms related to CVD, the 92 CVD-related human protein biomarkers of the Olink Multiplex Cardiovascular Disease II (CVDII) panel were measured using the Proximity Extension Assay (PEA) technology (Olink Biosciences, Uppsala, Sweden), as described elsewhere [3, 26]. In brief, once-thawed ethylenediaminetetraacetic acid (EDTA)-blood plasma was used for analysis. For each target antigen, the affinity-based PEA technique uses a pair of antibodies linked to unique, partially complementary single-stranded DNA oligonucleotides. After simultaneous binding of both antibodies to an antigen molecule, close proximity allows for the formation of a PCR target sequence by hybridization. After unspecific pre-amplification, amplicons were quantified by qPCR using protein-specific primer pairs. The resulting *C*_t_ value of each protein (Fluidigm Real-Time PCR Analysis Software, Version 4.3.1, San Francisco, USA) was transformed to normalized protein expression (NPX) units using software from the manufacturer (Olink^®^ NPX Manager, Version 1.1.4.0, Uppsala, Sweden). NPX units represent relative quantifications of protein concentrations on a log2-scale (i.e. an increase by 1 NPX represents a duplication of protein concentration). The investigation was performed for a subset of 22 individuals showing the greatest delta between FMD in control night and FMD after Noise 60.

### Statistical analysis

To analyze differences for primary and secondary outcomes, a repeated measures analysis of variance (ANOVA) was used, incorporating the three noise patterns as a fixed factor, first evaluating overall differences, then differences between each two out of three patterns. The significance level for primary and secondary endpoints was set to a two-sided significance level of 5% without adjusting for the multiple testing for the secondary outcomes. Continuous data variables are presented as mean ± standard deviation. Kolmogorov–Smirnov test was used to assess whether the data were normally distributed.

The potential carryover effect (priming) between two noise levels was evaluated using the mixed model analysis including individuals as random effect and night noise level and noise exposition in the previous study night as the fixed effect variables in the model. Linear mixed models were used to analyze differences between noise and control nights, with adjustment for PSQI, overall noise sensitivity (NoiSeQ), sleep-related noise sensitivity, attitude toward train noise, and morningness–eveningness questionnaire (MEQ).

An interim analysis was performed after enrolment of 70 participants as foreseen in study protocol. The study was ended after delivering statistically unambiguous answer to the primary question (Peto limit *p* < 0.001). For statistical evaluation of the proteomic data, paired *t* tests were used for each biomarker, or a Wilcoxon signed ranks test, respectively, when the normality assumption of the differences was violated. Statistical analysis was performed using IBM SPSS Statistics Version 23 and SAS Version 9.4. However, due to the high number of biomarkers in comparison to the limited number of noise exposures assessed by targeted proteomics, the correlation between protein biomarkers and skewed distributions may limit the usefulness of this classical statistical approach. To overcome these potential limitations of biomarker selection in a multi-variable model, we applied a supervised machine learning method based on a conditional logistic regression model with Least Absolute Shrinkage and Selection Operator (LASSO) penalties for variable selection [43]. A fourfold cross validation was applied for lambda.

### Database search

STRING (Search Tool for the Retrieval of Interacting Genes) version 11.0 [55] is a biological database and web resource providing information from multiple resources including text mining on known and predicted protein–protein interactions of more than 24 million proteins. To identify interactive relationships among identified target proteins, protein list was mapped to STRING.

## Results

### Functional and biochemical clinical parameters

**Fig. 1:**
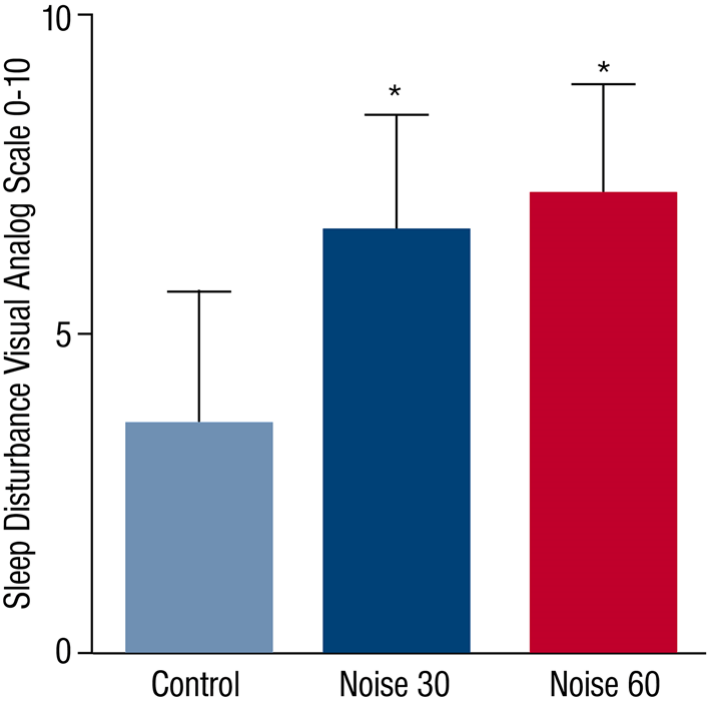
Effects of nighttime train noise on sleep disturbance. The Sleep Disturbance Visual Analog Scale 0–10 (VAS 0–10) was applied on control, Noise30 and Noise60 study nights. Data are mean ± SD of 70 study nights

The characteristics of the study population are shown in suppl. Table S1. *L*_Aeq_, the average sound pressure level, was 33.32 ± 4.58 dB(A) during control nights, 52 ± 2.69 dB(A) during Noise30 nights and 54.45 ± 2.6 dB(A) during Noise60 nights (Table 1). Peak levels of noise were lowest during control nights, whereas there were no differences between Noise30 and Noise60 nights (Table 1). Sleep quality (Visual Analog Scale 0–10) was significantly impaired after both noise patterns (Table 1, Fig. 1).

**Table 1:** Effects of nocturnal train noise on sleep disturbance, hemodynamic parameters, laboratory parameters, catecholamines

	Control	Noise30	Noise60	*p* (ANOVA)
Peak dB(A)	64.63 ± 8.62	74.9 ± 3.56	74.49 ± 4.02	< 0.001
*L*_Aeq_ dB(A)	33.32 ± 4.58	52 ± 2.69	54.45 ± 2.6	< 0.001
Sleep disturbance (VAS 0–10)	3.6 ± 2.06	6.62 ± 1.8	7.19 ± 1.71	< 0.001
Hemodynamic parameter				
HR mean	59.5 ± 8.1	58.7 ± 8.2	59.6 ± 8.4	0.377
HR max	104.6 ± 14.2	106.5 ± 16.8	107.3 ± 12.6	0.283
HR accel index	155.1 ± 144.2	177.8 ± 176.1	168.4 ± 146.5	0.843
BPsyst mean	115.3 ± 13.8	116.9 ± 13.5	114.1 ± 13.9	0.294
BPdiast mean	72.90 ± 11	74.10 ± 10.4	72.7 ± 10.1	0.475
BP rise index	31.1 ± 39.7	30.8 ± 31.3	38.8 ± 45.4	0.879
PTTmean	333.7 ± 19.2	332.5 ± 24.8	332.7 ± 19.8	0.377
PTTmax	373.2 ± 43.2	380 ± 19.2	375.3 ± 45.4	0.641
PTTmin	281.6 ± 26.2	274.1 ± 27.2	272.9 ± 26.5	0.088
Laboratory parameters				
CRP (mg/l)	2.00 ± 7.73	1.96 ± 7.91	1.13 ± 1.89	0.969
Neutrophils (%)	52 ± 8.7	52.5 ± 9.0	52.8 ± 8.4	0.626
Cortisol (μg/l)	15.46 ± 5.11	15.55 ± 5.4	15.15 ± 4.42	0.519
Glucose (mg/dl)	86.8 ± 6.2	86.5 ± 6.6	88 ± 6.4	0.058
Adrenalin (pg/ml)	25.6 ± 22.0	23.0 ± 18.18	25.9 ± 21.6	0.295
Noradrenalin (pg/ml)	144.4 ± 109.7	144.6 ± 123.3	157.6 ± 113.6	0.570
Dopamine (pg/ml)	10.07 ± 10.8	9.02 ± 8.8	10.68 ± 9.2	0.506
8-Isoprostan (pg/ml)	39.1 ± 20	40.5 ± 22.3	40.1 ± 20.4	0.697

Data are presented as mean ± SD

*L*_*Aeq*_, *dB* long-term equivalent continuous sound level, *PTT* pulse transit time, *BP* blood pressure, *HR accel index* heart rate acceleration index

**Fig. 2:**
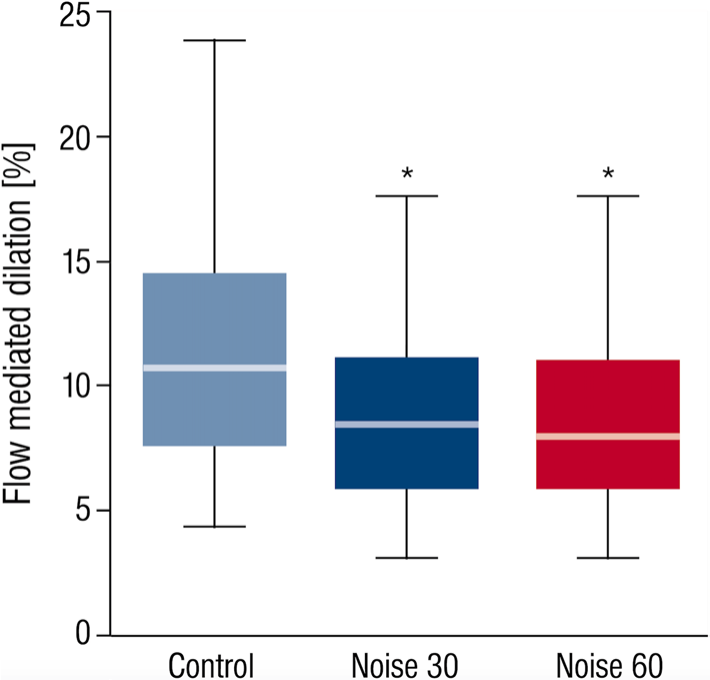
Endothelial function measurement by flow-mediated dilation upon train noise exposure. FMD was determined for control, Noise30 and Noise60 study nights. Exposure to both train noise patterns impaired endothelial function, although no difference was observed between Noise30 and Noise60 study nights. Data are mean ± SD of 69 (Noise30 and Noise60) or 70 (control) individual study nights in a randomized crossover fashion. *p* < 0.001. Box plots indicate minimum, maximum, 25% interquartile, median and 75% interquartile

**Fig. 3:**
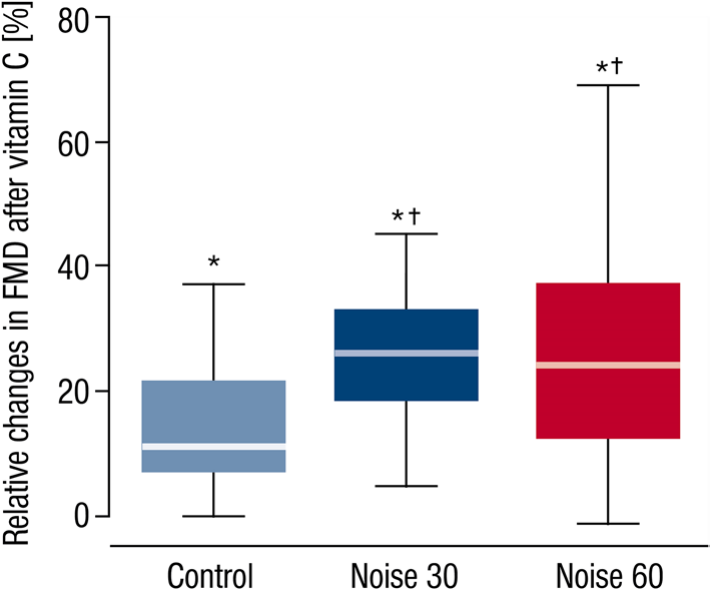
Determination of the effect of vitamin C on endothelial function changes by train noise exposure. FMD was determined for control, Noise30 and Noise60 study nights prior and post-administration of vitamin C, which was used as an antioxidant drug to assess the impact of noise-triggered oxidative stress on endothelial function. Vitamin C significantly improved FMD in all study groups (asterisk) being significantly stronger in nighttime railway noise-exposed study participants (dagger) *p *< 0.001. Box plots indicate minimum, maximum, 25% interquartile, median and 75% interquartile

In line with these data, the primary endpoint endothelial function was significantly impaired by both noise exposure scenarios with mean FMD levels of 11.23 ± 4.68% after control nights, 8.71 ± 3.83% after Noise30 nights and 8.47 ± 3.73% after Noise60 nights (Fig. 2). Post hoc analyses showed a significant difference between the control night and both noise exposure scenarios, whereas there was no significant difference between the two noise scenarios. Administration of vitamin C improved FMD for all three exposure nights (Control, Noise30, Noise60). The percent increase of FMD after Noise30 and Noise60 nights was significantly higher than the percent increase after a Control night (Fig. 3), indicating a higher degree of oxidative stress within the vasculature. Percent increase of FMD after Vitamin C intake was 16.67 ± 15.99% for control, 27.84 ± 17.77% for Noise30 and 29.22 ± 24.12% for Noise60 (*p *= 0.011).

No association was seen between noise-induced alteration of the participants’ FMD and overall noise sensitivity, sleep-related noise sensitivity or attitude toward train noise (results not shown). None of the hemodynamic (heart rate, blood pressure, pulse transit time) or laboratory parameters (inflammation: CRP, neutrophils; stress hormones: cortisol, catecholamines; oxidative stress: 8-isoprostane; metabolism: glucose) changed significantly, but the HRmax followed the expected trend of higher values after noise (Table 1). There were no significant changes concerning electrocardiogram recordings during the study nights (results not shown). Noise-induced blunting of FMD was not influenced by the randomization sequence, confirming no carryover effect (results not shown).

### Proteomic analysis of plasma proteins and protein database search

**Fig. 4:**
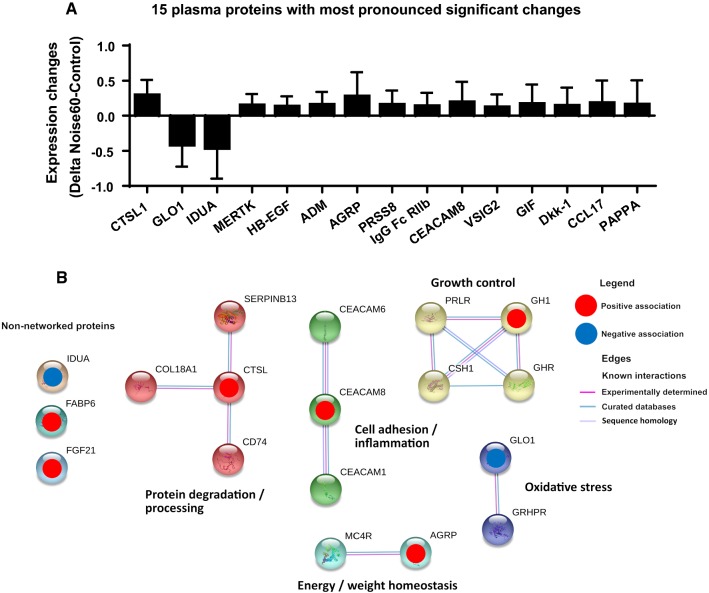
Changes of the plasma proteome upon train noise exposure. **a** 92 CVD-related human protein biomarkers were measured for control and Noise60 study nights by PEA technology. Exposure to Noise60 caused substantial changes in the plasma proteome as revealed by a total of 31 significantly changed targets. Here, the 15 plasma proteins with most pronounced significant changes are shown as revealed by paired *t* test analysis of each target prior/post-noise exposure. STRING database protein–protein interaction analysis of proteins selected by significant changes in *t* test analysis is shown in suppl. Figure S2. **b** STRING-database protein–protein interaction analysis of proteins selected by LASSO-regularized logistic regression revealing changes in protein pathways/clusters centered on growth control, oxidative stress, cell adhesion/inflammation, protein degradation/processing as well as some non-networked proteins. Maximal number of interactions to show 1st shell: 10. The non‐networked proteins shown in this figure are sorted by strength of evidence, which is based on their consistency of selection across both LASSO‐regularized regression analyses (highest evidence) and lambda ratios. Node colors indicate cluster membership, as determined using an unsupervised three inflation parameter Markov clustering algorithm. All measured targets are shown in suppl. Table S2. A targeted proteomic analysis was performed for 22 individuals showing the greatest delta between FMD in control night and FMD after Noise60

Paired *t* test-based statistical analysis of the proteomic expression signatures of the 92 plasma proteins revealed significant noise-related changes of 31 targets (for expression changes of all 92 targets see suppl. Table S2). The 15 proteins with the most pronounced significant changes are shown in Fig. 4a. A brief description of the biological functions of all significantly changed proteins is shown in suppl. Table S3. The statistical assessment of noise-associated protein signatures utilizing LASSO-regularized logistic regression supervised machine learning, however, revealed eight independently noise-regulated proteins (downregulated: GLO1, IDUA; upregulated: CTSL1, AGRP, CEACAM8, GT, FGF-21, GH) (Fig. 4b).

A bioinformatic analysis of protein–protein interactions and functional clusters of these 31 proteins utilizing STRING setting the minimum required interaction score to medium confidence (0.400), extending the protein nodes beyond identified proteins based on this selection criterion and including MCL clustering with 3 inflation parameters, revealed that the majority of the significantly changed proteins can be assigned to (patho)physiologically relevant functional clusters (suppl. Figure S2). In detail, upregulated proheparin-binding EGF-like growth factor (HBEGF) and angiopoietin-1 receptor (TEK) form a potential signaling hub with angiopoietins impacting angiogenesis, vascular stability and heart function in part by recruitment of vascular smooth muscle cells by endothelial cells [20]; upregulated tissue factor (F3), proteinase-activated receptor 1 (F2R), thrombomodulin (THBD) and downregulated alpha-L-iduronidase (IDUA) form a potential signaling hub with coagulation factors FII, FV, FVII, fibrinogen and serpins C1/D1 with important functions in hemostasis and coagulation; upregulated manganese superoxide dismutase (SOD2) and downregulated lactoylglutathione lyase (GLO1) are potentially associated with SOD1, catalase and hydroxyacylglutathione hydrolase (HAGH) in oxidative stress and glucotoxicity response; upregulated adrenomedullin (ADM) may interact with calcitonin-gene-related-peptide-receptor (CALCLR) in regulation of blood pressure, fluid and electrolyte homeostasis; upregulated gastric intrinsic factor (GIF) potentially interacts with cubilin (CUBN), hepatitis A virus cellular receptor 1 (HAVCR1) and low-density lipoprotein-related protein 2 (LRP2) in lipoprotein, vitamin, and iron metabolism; upregulated Dickkopf-related protein 1 (DKK1) forms a cluster with proto-oncogene protein (WNT1) and low-density lipoprotein receptor-related protein 6 (LRP6) in inflammation, CVD and bone development; upregulated tumor necrosis factor receptor superfamily member 11A (TNFRSF13A) and osteoclast-associated immunoglobulin-like receptor (OSCAR) are associated with TNFRSF13B and tumor necrosis factor ligand superfamily member 11 (TNFSF11) in osteoclastogenesis, T-cell-dendritic cell interactions; upregulated Low affinity immunoglobulin gamma Fc region receptor II-b (FCGR2B), C–C motif chemokine 17 (CCL17) and programmed cell death 1 ligand 2 (PDCD1LG2) form a cluster with programmed cell death protein 1 (PDCD1) and programmed cell death 1 ligand 1 (CD274) around T-cell proliferation and T- and B-cell function. The full names of all measured targets and their biological functions can be found in suppl. Table S3. The STRING analysis of the 8 proteins selected by LASSO setting the minimum required interaction score to highest confidence (0.900) and the maximal number of first shell interactors to no more than 10, and including MCL clustering with 3 inflation parameters, confirmed pathways/clusters centered on growth control, oxidative stress, cell adhesion/inflammation, protein degradation/processing as well as 3 non-networked proteins (Fig. 4b).

## Discussion

In the present study, we found that nighttime railway noise exposure was associated with endothelial dysfunction, quantified using FMD measurements. Noise30 and Noise60 resulted in a comparable impairment of FMD. Importantly, the study was stopped after enrolment of 70 participants as foreseen in study protocol, since the interim analysis was delivering the statistically unambiguous answer to the primary question of the present study. Administration of vitamin C leads to a stronger improvement of endothelial dysfunction after noise-exposed nights compared to the control night. The adverse effects on endothelial function by train noise were associated with an increase in sleep disturbance and a marked pro-thromboinflammatory phenotype.

According to the WHO Environmental Noise Guidelines for the European Region, railway noise may increase the risk for ischemic heart disease, whereas the data are less clear with respect to hypertension, stroke or diabetes risk [22]. A recent Swiss study concluded that years of life lost due to traffic are dominated by air pollution, whereas morbidity and quality of life are dominated by noise [57]. Only limited research on acute effects of traffic noise exposure on vascular (endothelial) function parameters exists. Numerous clinical studies have investigated endothelial function in subjects with cardiovascular disease and established the prognostic importance of coronary and peripheral endothelial dysfunction, not only for patients with CAD [44], peripheral arterial occlusive disease [2] arterial hypertension [40], postmenopausal women [28] and heart failure [16], but also in healthy subjects [27, 51]. Previously we have demonstrated that acute nighttime aircraft noise exposure (30 and 60 nighttime events) tended to worsen endothelial function (impaired FMD) in healthy volunteers [46] and resulted in significant endothelial dysfunction in patients with already established CAD or cardiovascular risk factors [45].

With the present study, we found a highly significant impairment of endothelial function in healthy subjects in response to noise scenarios of both 30 and 60 nighttime train events. A potential reason as to why we find stronger effect of exposure to events of railway noise as compared to aircraft noise on endothelial function could be the levels of sound pressure in the two studies, with peaks of 73–75 dB(A) and a mean of 52–54 dB(A) in the train study and peaks of 60 dB(A) and means of 43–46 dB(A) in the aircraft study. Impaired endothelial function was associated with impaired sleep quality of the exposed subjects. Importantly, sleep fragmentation and too short sleep (deprivation) are well-known triggers of endothelial dysfunction and facilitate the development of cardiovascular disease and increases in overall mortality [64, 65]. Previously, we demonstrated in healthy subjects and patients with coronary artery disease that nighttime aircraft noise exposure to 30 or 60 flight events caused a substantial increase in sleep disturbance and FMD [45, 46]. Of note, we do not consider impaired sleep quality a confounder of noise effects on FMD but a direct consequence of noise exposure. Interestingly, others found that daily sleep deprivation for 2 h/day for 8 days induces endothelial dysfunction in healthy subjects, and the degree of endothelial dysfunction was comparable to that observed in 24 h shift-workers [1] and in humans exposed to chronic sleep restriction [56]. A study in mice reported endothelial dysfunction, arterial hypertension, vascular inflammation and senescence upon exposure to 20 weeks of sleep deprivation [9]. In addition, activation of NADPH oxidase [35] and increased oxidative stress [58] were reported for mice subjected to sleep fragmentation. We recently showed that genetic Nox2 deletion prevents aircraft noise-mediated cardiovascular and cerebral damage by reducing inflammation and oxidative stress [23].

As part of the most recent WHO environmental noise guidelines for the European region, a meta-analysis of psychoacoustic surveys on self-reported sleep disturbance (percent highly disturbed) showed significantly impaired sleep quality for aircraft (1.9%), road (2.1%) and rail (3.1%) per 10 dB(A) increase in noise [7], indicating that any source of nocturnal traffic noise impairs sleep quality. These results correlate well with previously demonstrated impaired sleep quality caused by aircraft noise in humans, which was also associated with impaired endothelial function [45, 46]. In addition, there is a significant association between nighttime (but not daytime) aircraft noise and prevalent hypertension within the HYENA cohort (5000 persons living near one of six major European airports) [21] and between nighttime but not daytime traffic noise and increased vascular stiffness [13].

Previously we have shown in a very small group of healthy subjects (*n* = 5) that vitamin C treatment in subjects exposed to nighttime aircraft noise significantly improved FMD [46]. In the present study, we randomly assigned persons sleeping with and without the noise scenarios to acute vitamin C challenges and observed a significantly stronger improvement in FMD in subjects after noise exposure nights compared to control nights. This observation strongly suggests that increased production of reactive oxygen species represents an important mechanism underlying endothelial dysfunction function supported by vitamin C displaying a stronger antioxidant effect in the presence of a higher burden of oxidative stress in the noise exposure groups.

This observation is in line with our previous experimental study of aircraft noise exposure [23, 29], supporting that nighttime noise exposure is able to increase vascular and cerebral oxidative stress in mice via activation of the phagocytic NADPH oxidase (NOX2) and by uncoupling of the nitric oxide synthase (endothelial and neuronal type NOS). The demonstration of increased oxidative stress within the vasculature has also important prognostic implications, since we have previously shown that a more pronounced improvement of endothelial function by vitamin C was associated with a worse prognosis, most probably due to higher oxidative coronary stress burden in the individuals with enhanced vitamin C effects [17].

When evaluating a subset of 22 individuals with the most pronounced noise-triggered changes in FMD, we established a substantial regulation of the plasma proteome (and related pathways therein) by nighttime train noise exposure. The detailed proteomic evaluation of the (patho)physiological status, analyzing interactions and functional clusters of the proteins altered by noise exposure (Fig. 4), revealed an acutely prooxidative, pro-thrombotic, and proinflammatory phenotype in response to transient train noise exposure. This is in accordance with previous animal data on stimulation of inflammatory and oxidative stress pathways by noise exposure [23, 29, 43]. This phenotype is likely further marked by alterations in cardiovascular homeostasis, metabolic control, and immune response, which is basically in accordance with previous animal data on stimulation of inflammatory and oxidative stress pathways by noise exposure [23, 29, 43]. For in-depth analysis of the proteomic data, see extended discussion in the online supplement.

### Strengths and limitations of the study

This study was designed as a field study with minimal sleep disruption due to environment and equipment, thus creating ecologically valid conditions. Thus, a pure laboratory environment, where ambient conditions, sound levels, and external stimuli can be controlled at the expense of creating artificial rather than familiar conditions, was avoided. This is a strength as sleep is known to be very sensitive to changes in surroundings and study subjects usually show more alterations in sleep in the laboratory than in the field.

A limitation of the present study was that many measurements as well as blood sampling are not conducted directly after awakening—instead people had to commute to the study center, which could potentially expose them to a number of different pollutants, e.g. noise and air pollution, that may diluted the effect. We tried to overcome this limitation by analyzing impact of travel distance on the measured parameters and found no significant changes in the median split analysis (not shown). However, travel distance only partially reflects the travel time (delay between awakening and blood sample collection or measurement of parameters) and allows only rough estimation of confounding exposures during travel such as noise, other mental stress or air pollution, all of which may influence the outcome of measurements. Stress hormone levels are known to show large variations over the day and, thus, the estimation of this parameter hours after awakening may not have been optimal. Future studies should collect samples immediately after awakening, e.g. collecting morning saliva, which is recognized as good media for measurement of cortisol levels [50].

A limitation of the present study was that blood cell composition as well as platelet activity before and after noise exposure was not evaluated. The design of the vitamin C substudy and the overall study protocol did only include measurement of FMD but no other parameters after vitamin C administration since this would have been a logistic challenge as well as a burden for the subjects when undergoing a second set of all measurements on the same day. Also our proteomic data do not allow discrimination between increased degradation or decreased expressional/translational changes of the regulated target proteins.

Another limitation of the present study was that we had no other noise source included with a similar mean sound pressure level (e.g. music) to elucidate whether traffic noise has a specific and unique impact on sleep quality and impairment of endothelial function. Likewise, we could have included a sleep fragmentation/deprivation control group with repeated wakeup calls.

## Conclusions and clinical implications

In the present study, we showed for the first time in a field study that nighttime railway noise is associated with a significant degree of endothelial dysfunction in healthy subjects. We also showed that treatment with vitamin C improved endothelial function predominately after nights with exposure to railway noise, compatible with a noise-induced increase in oxidative stress within the vasculature. The observed proteomic changes in response to nighttime railway noise point toward a prooxidative, proinflammatory and pro-thrombotic phenotype, providing a molecular basis to explain the increased cardiovascular risk observed in epidemiological noise studies. Interestingly, a literature search for transcriptional regulation of the eight target proteins identified by LASSO-based analysis revealed involvement of forkhead-type transcription factors, mainly FOXO1, in CTSL1, AGRP and GH regulation along with circadian control of AGRP, FGF-21 and GH expression. NFκB is involved in the regulation of GH and GLO1 and the latter was further controlled by NRF2. These transcriptional regulation data support the here and previously proposed prooxidative and proinflammatory milieu in response to noise exposure [29] and the central role of FOXO/circadian pathways is nicely mirrored by our previous transcriptome analysis in mice showing substantial dysregulation of circadian clock pathways with a role of FOXO3 [23]. These findings may have important public health implications since the number of people exposed to high levels of nighttime railway noise is increasing in Europe, with a rise in number of nightly freight trains. Thus, noise mitigation strategies including noise protection walls are important to protect people living close to highly trafficked railway tracks from cardiovascular adverse effects.

## Electronic supplementary material

Below is the link to the electronic supplementary material.

Supplementary material 1 (PDF 521 kb)
