# Supplementary material for: Acute exposure to nocturnal train noise induces endothelial dysfunction and pro-thromboinflammatory changes of the plasma proteome in healthy subjects
Source: Basic Res Cardiol. 2019 Oct 29;114(6):46. doi: 10.1007/s00395-019-0753-y (PMC6817813; doi:10.1007/s00395-019-0753-y)
Supplement: Supplementary file 1 — Supplementary material 1 (PDF 521 kb) [file 395_2019_753_MOESM1_ESM.pdf]

Online supplemental data

Acute exposure to nocturnal train noise induces endothelial dysfunction and pro-thromboinflammatory changes of the plasma proteome in healthy subjects

Johannes Herzog[1]*, Frank P. Schmidt[1]*, Omar Hahad[1], Seyed H. Mahmoudpour[2,3], Alina K. Mangold[1], Pascal Garcia Andreo[1], Jürgen Prochaska[3,4], Thomas Koeck[3,5], Philipp S. Wild[3,4,5], Mette Sørensen[6,7], Andreas Daiber[1,5], Thomas Münzel[1,3,5]

From the [1] Center for Cardiology, Cardiology I – Laboratory of Molecular Cardiology, University Medical Center of the Johannes Gutenberg-University Mainz, Mainz, Germany; [2] Institute for Medical Biostatistics, Epidemiology, and Informatics (IMBEI), Department of Biometry and Bioinformatics, University Medical Center of the Johannes Gutenberg University, Mainz, Germany; [3] Center for Thrombosis and Hemostasis, University Medical Center of the Johannes Gutenberg University Mainz, Mainz, Germany; [4] Preventive Cardiology and Preventive Medicine, Center for Cardiology, University Medical Center of the Johannes Gutenberg-University Mainz, Mainz, Germany; [5] German Center for Cardiovascular Research (DZHK), Partner Site Rhine-Main, Mainz, Germany. [6] Danish Cancer Society, Copenhagen, Denmark; [7] Department of Natural Science and Environment, Roskilde University, Roskilde, Denmark

Suppl. Table S1 Baseline characteristics of the study population.

Characteristics	Unit	Mean ± SD<br>(min – max)
**Age**	Year	25.74 ± 5.54<br>(19 - 48)
**Gender**	% Female	50
**Height**	cm	174.71 ± 9.89<br>(159 - 198)
**Weight**	kg	68.50 ± 11.99<br>(49 - 100)
**BMI**	kg/m^2	22.32 ± 2.59<br>(17.24 - 28.90)
**Baseline noise sensitivity, sleep quality index**		
**NoiSeQ**		1.26 ± 0.37<br>(0.34 - 2.31)
**PSQI**		5.29 ± 1.89<br>(0 – 10)
**Horne-Östberg**		51.22 ± 9.1<br>(34 – 71)
**Laboratory values**		
**C-reactive protein**	mg/L	1.31 ± 2.21<br>(0 – 14)
**Total cholesterol**	mg/dL	177.41 ± 30.25<br>(119 – 249)
**LDL**	mg/dL	96.66 ± 26.02<br>(51 – 165)
**HDL**	mg/dL	61.84 ± 12.56<br>(38 – 92)
**Triglycerides**	mg/dL	94.64 ± 43.38<br>(35 – 228)
**Creatinin**	mg/dL	0.86 ± 0.15<br>(0.63 – 1.39)
**HbA1C**	%	5.05 ± 0.30<br>(4.3 – 5.8)

Data are presented as mean ±SD and the (minimum-maximum).
NoiSeQ: Dortmund Noise Sensitivity Questionnaire with a score of three representing greatest noise sensitivity.
PSQI: Pittsburgh Sleep Quality Index with a higher score indicating worse sleep quality.
Horne-Östberg Morningness-Eveningness Questionnaire.

Suppl. Table S2. Data from targeted proteomics. Expression differences between Noise60 and Control exposure patterns are shown for all protein targets (92 biomarkers of cardiovascular disease). Proteomic expression analysis was performed for 22 individuals showing the greatest delta between FMD in control night and FMD after Noise60. The targets marked with green color were significantly changed as revealed by paired t-test when normally distributed or by Wilcoxon signed ranks test when normal distribution test failed (indicated by "W" in the last column and red color).

	Paired Differences							
				95% Confidence Interval				
Protein Target	Mean value (Delta Noise60-Control)	SD	SEM	Lower	Upper	T	df	Sig. (2-sided)
CTSL1_60 - CTSL1	0,29366682	0,21711056	0,04628813	0,19740539	0,38992825	6,344	21	0,000
GLO1_60 - GLO1	-0,41561500	0,31155399	0,06642353	-0,55375030	-0,27747970	-6,257	21	0,000
PD-L2_60 - PD-L2	0,11553182	0,10395732	0,02216378	0,06943972	0,16162391	5,213	21	0,000
IDUA_60 - IDUA	-0,46226591	0,43503992	0,09275082	-0,65515180	-0,26938001	-4,984	21	0,000
TF_60 - TF	0,10013136	0,10164091	0,02166991	0,05506631	0,14519642	4,621	21	0,000
MERTK_60 - MERTK	0,15043500	0,15936788	0,03397735	0,07977524	0,22109476	4,428	21	0,000
HB-EGF_60 - HB-EGF	0,13380500	0,14253422	0,03038840	0,07060887	0,19700113	4,403	21	0,000
ADM_60 - ADM	0,15896409	0,18085129	0,03855762	0,07877912	0,23914906	4,123	21	0,000
SOD2_60 - SOD2	0,04438091	0,05452573	0,01162493	0,02020555	0,06855627	3,818	21	0,001
AGRP_60 - AGRP	0,27577227	0,34446445	0,07344007	0,12304529	0,42849926	3,755	21	0,001
PRSS8_60 - PRSS8	0,15703773	0,20109344	0,04287327	0,06787789	0,24619757	3,663	21	0,001
TM_60 - TM	0,09336409	0,12368876	0,02637053	0,03852357	0,14820462	3,540	21	0,005 (W)
CEACAM8_60 - CEACAM8	0,19455000	0,28756309	0,06130866	0,06705167	0,32204833	3,173	21	0,005
VSIG2_60 - VSIG2	0,12153227	0,18205310	0,03881385	0,04081445	0,20225009	3,131	21	0,005
DCN_60 - DCN	0,08387273	0,12591398	0,02684495	0,02804560	0,13969986	3,124	21	0,005
IgG Fc receptor II-b_60 - IgG Fc receptor II-b	0,13819273	0,18903899	0,04030325	0,05437753	0,22200792	3,429	21	0,006 (W)
IL-4RA_60 - IL-4RA	0,09467818	0,25879256	0,05517476	-0,02006401	0,20942038	1,716	21	0,007 (W)

GIF_60 - GIF	0,16996227	0,27483550	0,05859513	0,04810704	0,29181751	2,901	21	0,009
SORT1_60 - SORT1	0,07646227	0,12378729	0,02639154	0,02157806	0,13134648	2,897	21	0,009
hOSCAR_60 - hOSCAR	0,05621591	0,11195103	0,02386804	0,00657960	0,10585222	2,355	21	0,010 (W)
TIE2_60 - TIE2	0,07631500	0,12781646	0,02725056	0,01964436	0,13298564	2,800	21	0,011
CD84_60 - CD84	0,10897864	0,18417228	0,03926566	0,02732122	0,19063605	2,775	21	0,011
Dkk-1_60 - Dkk-1	0,14512273	0,25433077	0,05422350	0,03235878	0,25788667	2,676	21	0,014
CCL17_60 - CCL17	0,18217182	0,31943855	0,06810453	0,04054070	0,32380294	2,675	21	0,014
PRSS27_60 - PRSS27	0,08086909	0,14760615	0,03146974	0,01542419	0,14631399	2,570	21	0,018
AMBP_60 - AMBP	0,05423909	0,10836574	0,02310365	0,00619241	0,10228577	2,348	21	0,029
TNFRSF11A_60 - TNFRSF11A	0,10201818	0,21484087	0,04580423	0,00676308	0,19727329	2,227	21	0,037
PAPPA_60 - PAPPA	0,16169364	0,34254896	0,07303168	0,00981594	0,31357134	2,214	21	0,038
IL1RL2_60 - IL1RL2	0,10343636	0,22650408	0,04829083	0,00301008	0,20386265	2,142	21	0,044
PRELP_60 - PRELP	-0,04835227	0,10769959	0,02296163	-0,09610359	-0,00060095	-2,106	21	0,047
PAR-1_60 - PAR-1	0,07839455	0,17473714	0,03725408	0,00092044	0,15586865	2,104	21	0,048
KIM1_60 - KIM1	0,11572636	0,20637984	0,04400033	0,02422267	0,20723006	2,630	21	0,053 (W)
MMP12_60 - MMP12	0,14569455	0,33486179	0,07139277	-0,00277486	0,29416395	2,041	21	0,054
PARP-1_60 - PARP-1	0,14474364	0,34160140	0,07282966	-0,00671394	0,29620121	1,987	21	0,060
ACE2_60 - ACE2	0,11357091	0,27205678	0,05800270	-0,00705231	0,23419413	1,958	21	0,064
Gal-9_60 - Gal-9	0,03623955	0,28443889	0,06064258	-0,08987360	0,16235269	0,598	21	0,067 (W)
TRAIL-R2_60 - TRAIL-R2	0,06021545	0,15559152	0,03317222	-0,00876996	0,12920087	1,815	21	0,084
PIgR_60 - PIgR	0,02220636	0,05822168	0,01241290	-0,00360768	0,04802041	1,789	21	0,088
IL6_60 - IL6	-0,27132227	0,72864857	0,15534840	-0,59438695	0,05174240	-1,747	21	0,095
PGF_60 - PGF	0,07957864	0,21683187	0,04622871	-0,01655923	0,17571650	1,721	21	0,100
BMP-6_60 - BMP-6	0,09388227	0,25866179	0,05514688	-0,02080194	0,20856649	1,702	21	0,103
GDF-2_60 - GDF-2	0,12622091	0,34870592	0,07434435	-0,02838663	0,28082845	1,698	21	0,104
THPO_60 - THPO	0,06407909	0,19786018	0,04218393	-0,02364720	0,15180538	1,519	21	0,144

LEP_60 - LEP	0,19120636	0,46167666	0,09842980	-0,01348960	0,39590233	1,943	21	0,149 (W)
GT_60 - GT	0,26428682	0,83407192	0,17782473	-0,10551995	0,63409359	1,486	21	0,152
CTRC_60 - CTRC	0,06875318	0,26534456	0,05657165	-0,04889401	0,18640037	1,215	21	0,158 (W)
STK4_60 - STK4	-0,23278864	0,77599278	0,16544222	-0,57684456	0,11126729	-1,407	21	0,174
LPL_60 - LPL	-0,11219455	0,37414825	0,07976868	-0,27808259	0,05369350	-1,406	21	0,174
FGF-23_60 - FGF-23	0,11162500	0,40151346	0,08560296	-0,06639610	0,28964610	1,304	21	0,206
CCL3_60 - CCL3	0,07645682	0,48829709	0,10410529	-0,14004198	0,29295562	0,734	21	0,211 (W)
BOC_60 - BOC	0,04712273	0,17430032	0,03716095	-0,03015771	0,12440316	1,268	21	0,219
DECR1_60 - DECR1	-0,14620273	0,54815381	0,11686678	-0,38924051	0,09683506	-1,251	21	0,225
IL-17D_60 - IL-17D	0,04752409	0,19542480	0,04166471	-0,03912241	0,13417059	1,141	21	0,267
XCL1_60 - XCL1	-0,06588864	0,28774288	0,06134699	-0,19346668	0,06168941	-1,074	21	0,295
TGM2_60 - TGM2	0,12167500	0,54489207	0,11617138	-0,11991661	0,36326661	1,047	21	0,307
VEGFD_60 - VEGFD	0,04623636	0,21160085	0,04511345	-0,04758220	0,14005493	1,025	21	0,317
FGF-21_60 - FGF-21	0,22549636	1,07414370	0,22900821	-0,25075227	0,70174500	0,985	21	0,336
TNFRSF10A_60 - TNFRSF10A	0,03874682	0,18756736	0,03998949	-0,04441589	0,12190952	0,969	21	0,344
FABP2_60 - FABP2	-0,13661045	0,67914612	0,14479444	-0,43772698	0,16450607	-0,943	21	0,356
SLAMF7_60 - SLAMF7	0,04617091	0,22997237	0,04903027	-0,05579313	0,14813495	0,942	21	0,357
IL18_60 - IL18	0,01003273	0,27929169	0,05954519	-0,11379827	0,13386372	0,168	21	0,390 (W)
CD4_60 - CD4	-0,04301136	0,23346843	0,04977564	-0,14652547	0,06050274	-0,864	21	0,397
TNFRSF13B_60 - TNFRSF13B	0,03930955	0,22684519	0,04836356	-0,06126798	0,13988707	0,813	21	0,425
ADAM-TS13_60 - ADAM-TS13	-0,04317409	0,17852140	0,03806089	-0,12232605	0,03597787	-1,134	21	0,426 (W)
IL-27_60 - IL-27	-0,04269364	0,24950594	0,05319484	-0,15331837	0,06793110	-0,803	21	0,431
MMP7_60 - MMP7	-0,13148364	0,76067169	0,16217575	-0,46874657	0,20577930	-0,811	21	0,445 (W)
SCF_60 - SCF	0,02649636	0,16041067	0,03419967	-0,04462574	0,09761847	0,775	21	0,447
SPON2_60 - SPON2	0,01989091	0,12592458	0,02684721	-0,03594092	0,07572274	0,741	21	0,467
LOX-1_60 - LOX-1	0,06317591	0,41212343	0,08786501	-0,11954938	0,24590120	0,719	21	0,480
REN_60 - REN	0,05931045	0,39064199	0,08328515	-0,11389050	0,23251141	0,712	21	0,484

FS_60 - FS	-0,08243318	0,54621845	0,11645416	-0,32461288	0,15974651	-0,708	21	0,487
PDGF subunit B_60 - PDGF subunit B	0,09537545	0,68574653	0,14620165	-0,20866753	0,39941843	0,652	21	0,521
BNP_60 - BNP	0,10513864	0,66002854	0,14071856	-0,18750162	0,39777890	0,747	21	0,527 (W)
NEMO_60 - NEMO	0,07107818	0,56994439	0,12151255	-0,18162100	0,32377737	0,585	21	0,565
PTX3_60 - PTX3	-0,03539045	0,29968371	0,06389278	-0,16826277	0,09748186	-0,554	21	0,585
SRC_60 - SRC	-0,08714091	0,74029883	0,15783224	-0,41537102	0,24108921	-0,552	21	0,587
IL16_60 - IL16	0,03241545	0,28150385	0,06001682	-0,09239636	0,15722727	0,540	21	0,595
IL-1ra_60 - IL-1ra	0,02121091	0,68285333	0,14558482	-0,28154930	0,32397111	0,146	21	0,615 (W)
PSGL-1_60 - PSGL-1	0,01127045	0,10651686	0,02270947	-0,03595648	0,05849738	0,496	21	0,625
MARCO_60 - MARCO	-0,01226864	0,12823137	0,02733902	-0,06912324	0,04458597	-0,449	21	0,658
CXCL1_60 - CXCL1	0,10684682	0,59368229	0,12657349	-0,15637716	0,37007080	0,844	21	0,685 (W)
SERPINA12_60 - SERPINA12	-0,05434682	0,63004729	0,13432653	-0,33369414	0,22500050	-0,405	21	0,690
RAGE_60 - RAGE	0,01414455	0,17067562	0,03638816	-0,06152878	0,08981788	0,389	21	0,701
ANG-1_60 - ANG-1	0,05107773	0,64801798	0,13815790	-0,23623735	0,33839280	0,370	21	0,715
HAOX1_60 - HAOX1	-0,27045955	1,35290970	0,28844132	-0,87030610	0,32938701	-0,938	21	0,733 (W)
THBS2_60 - THBS2	0,00869818	0,11963381	0,02550601	-0,04434448	0,06174084	0,341	21	0,736
CA5A_60 - CA5A	0,03702909	0,63302065	0,13496046	-0,24363654	0,31769472	0,274	21	0,786
GH_60 - GH	0,08957955	1,64655103	0,35104586	-0,64046028	0,81961937	0,255	21	0,801
HSP 27_60 - HSP 27	0,02335318	0,45677465	0,09738468	-0,17916935	0,22587571	0,240	21	0,813
HO-1_60 - HO-1	-0,00507591	0,20714439	0,04416333	-0,09691859	0,08676677	-0,115	21	0,910
ITGB1BP2_60 - ITGB1BP2	-0,00761773	0,50300008	0,10723998	-0,23063547	0,21540002	-0,071	21	0,944
CD40-L_60 - CD40-L	0,02655682	0,31364743	0,06686986	-0,11250666	0,16562030	0,397	21	0,961 (W)

Suppl. Table S3. Data from targeted proteomics. Brief functional and biochemical information on the 31 significantly changed protein targets as listed in **suppl. Table S2**.

DCN	*Decorin; May affect the rate of fibrils formation; Small leucine rich repeat proteoglycans (359 aa)*
CCL17	*C-C motif chemokine 17; Chemotactic factor for T-lymphocytes but not monocytes or granulocytes. May play a role in T-cell development in thymus and in trafficking and activation of mature T-cells. Binds to CCR4; Belongs to the intercrine beta (chemokine CC) family (94 aa)*
HBEGF	*Proheparin-binding EGF-like growth factor; Growth factor that mediates its effects via EGFR, ERBB2 and ERBB4. Required for normal cardiac valve formation and normal heart function. Promotes smooth muscle cell proliferation. May be involved in macrophage-mediated cellular proliferation. It is mitogenic for fibroblasts, but not endothelial cells. It is able to bind EGF receptor/EGFR with higher affinity than EGF itself and is a far more potent mitogen for smooth muscle cells than EGF. Also acts as a diphtheria toxin receptor (208 aa)*
CEACAM8	*Carcinoembryonic antigen related cell adhesion molecule family; Belongs to the immunoglobulin superfamily. CEA family (349 aa)*
IDUA	*Alpha-L-iduronidase; Iduronidase, alpha-L- (653 aa)*
SORT1	*Sortilin; Functions as a sorting receptor in the Golgi compartment and as a clearance receptor on the cell surface. Required for protein transport from the Golgi apparatus to the lysosomes by a pathway that is independent of the mannose-6-phosphate receptor (M6PR). Also required for protein transport from the Golgi apparatus to the endosomes. Promotes neuronal apoptosis by mediating endocytosis of the proapoptotic precursor forms of BDNF (proBDNF) and NGFB (proNGFB). Also acts as a receptor for neurotensin. May promote mineralization of the extracellular matrix during osteogenic differ [...] (831 aa)*
GIF	*Gastric intrinsic factor; Promotes absorption of the essential vitamin cobalamin (Cbl) in the ileum. After interaction with CUBN, the GIF-cobalamin complex is internalized via receptor-mediated endocytosis (417 aa)*
IL1RL2	*Interleukin-1 receptor-like 2; Receptor for interleukin-36 (IL36A, IL36B and IL36G). After binding to interleukin-36 associates with the coreceptor IL1RAP to form the interleukin-36 receptor complex which mediates interleukin-36-dependent activation of NF-kappa-B, MAPK and other pathways (By similarity). The IL-36 signaling system is thought to be present in epithelial barriers and to take part in local inflammatory response; it is similar to the IL-1 system. Seems to be involved in skin inflammatory response by induction of the IL- 23/IL-17/IL-22 pathway; Immunoglobulin like domain co [...] (575 aa)*

IL-4RA	*Interleukin-4 receptor subunit alpha; Receptor for both interleukin 4 and interleukin 13. Couples to the JAK1/2/3-STAT6 pathway. The IL4 response is involved in promoting Th2 differentiation. The IL4/IL13 responses are involved in regulating IgE production and, chemokine and mucus production at sites of allergic inflammation. In certain cell types, can signal through activation of insulin receptor substrates, IRS1/IRS2 (825 aa)*
AMBP	*Protein AMBP; Inter-alpha-trypsin inhibitor inhibits trypsin, plasmin, and lysosomal granulocytic elastase. Inhibits calcium oxalate crystallization; Lipocalins (352 aa)*
AGRP	*Agouti-related protein; Plays a role in weight homeostasis. Involved in the control of feeding behavior through the central melanocortin system. Acts as alpha melanocyte-stimulating hormone antagonist by inhibiting cAMP production mediated by stimulation of melanocortin receptors within the hypothalamus and adrenal gland. Has very low activity with MC5R (By similarity). Is an inverse agonist for MC3R and MC4R being able to suppress their constitutive activity. It promotes MC3R and MC4R endocytosis in an arrestin-dependent manner; Endogenous ligands (132 aa)*
MERTK	*Tyrosine-protein kinase Mer; Receptor tyrosine kinase that transduces signals from the extracellular matrix into the cytoplasm by binding to several ligands including LGALS3, TUB, TULP1 or GAS6. Regulates many physiological processes including cell survival, migration, differentiation, and phagocytosis of apoptotic cells (efferocytosis). Ligand binding at the cell surface induces autophosphorylation of MERTK on its intracellular domain that provides docking sites for downstream signaling molecules. Following activation by ligand, interacts with GRB2 or PLCG2 and induces phosphorylation [...] (999 aa)*
PRSS27	*Serine protease 27 (290 aa)*
CD84	*SLAM family member 5; Self-ligand receptor of the signaling lymphocytic activation molecule (SLAM) family. SLAM receptors triggered by homo- or heterotypic cell-cell interactions are modulating the activation and differentiation of a wide variety of immune cells and thus are involved in the regulation and interconnection of both innate and adaptive immune response. Activities are controlled by presence or absence of small cytoplasmic adapter proteins, SH2D1A/SAP and/or SH2D1B/EAT-2. Can mediate natural killer (NK) cell cytotoxicity dependent on SH2D1A and SH2D1B (By similarity). Increa [...] (345 aa)*
VSIG2	*V-set and immunoglobulin domain containing 2 (327 aa)*
PRSS8	*Prostasin; Possesses a trypsin-like cleavage specificity with a preference for poly-basic substrates. Stimulates epithelial sodium channel (ENaC) activity through activating cleavage of the gamma subunits (SCNN1G); Serine proteases (343 aa)*
F2R	*Proteinase-activated receptor 1; High affinity receptor for activated thrombin coupled to G proteins that stimulate*

	phosphoinositide hydrolysis. May play a role in platelets activation and in vascular development; F2R receptors (425 aa)
PAPPA	*Pappalysin-1; Metalloproteinase which specifically cleaves IGFBP-4 and IGFBP-5, resulting in release of bound IGF. Cleavage of IGFBP-4 is dramatically enhanced by the presence of IGF, whereas cleavage of IGFBP-5 is slightly inhibited by the presence of IGF; Belongs to the peptidase M43B family (1627 aa)*
F3	*Tissue factor; Initiates blood coagulation by forming a complex with circulating factor VII or VIIa. The [TF-VIIa] complex activates factors IX or X by specific limited protolysis. TF plays a role in normal hemostasis by initiating the cell-surface assembly and propagation of the coagulation protease cascade (295 aa)*
PRELP	*Prolargin; May anchor basement membranes to the underlying connective tissue; Small leucine rich repeat proteoglycans (382 aa)*
HAVCR1	*Hepatitis A virus cellular receptor 1; May play a role in T-helper cell development and the regulation of asthma and allergic diseases. Receptor for TIMD4 (By similarity). May play a role in kidney injury and repair; Belongs to the immunoglobulin superfamily. TIM family (364 aa)*
CTSL	*Cathepsin L1; Important for the overall degradation of proteins in lysosomes; Cathepsins (333 aa)*
FCGR2B	*Low affinity immunoglobulin gamma Fc region receptor II-b; Receptor for the Fc region of complexed or aggregated immunoglobulins gamma. Low affinity receptor. Involved in a variety of effector and regulatory functions such as phagocytosis of immune complexes and modulation of antibody production by B- cells. Binding to this receptor results in down-modulation of previous state of cell activation triggered via antigen receptors on B-cells (BCR), T-cells (TCR) or via another Fc receptor. Isoform IIB1 fails to mediate endocytosis or phagocytosis. Isoform IIB2 does not trigger phagocytosis [...] (310 aa)*
GLO1	*Lactoylglutathione lyase; Catalyzes the conversion of hemimercaptal, formed from methylglyoxal and glutathione, to S-lactoylglutathione. Involved in the regulation of TNF-induced transcriptional activity of NF- kappa-B. Required for normal osteoclastogenesis (184 aa)*
DKK1	*Dickkopf-related protein 1; Antagonizes canonical Wnt signaling by inhibiting LRP5/6 interaction with Wnt and by forming a ternary complex with the transmembrane protein KREMEN that promotes internalization of LRP5/6. DKKs play an important role in vertebrate development, where they locally inhibit Wnt regulated processes such as antero-posterior axial patterning, limb development, somitogenesis and eye formation. In the adult, Dkks are implicated in bone formation and bone disease, cancer and Alzheimer disease. Inhibits the pro-apoptotic function of KREMEN1 in a Wnt-independent manner [...] (266 aa)*

THBD	*Thrombomodulin; Thrombomodulin is a specific endothelial cell receptor that forms a 1-1 stoichiometric complex with thrombin. This complex is responsible for the conversion of protein C to the activated protein C (protein Ca). Once evolved, protein Ca scissions the activated cofactors of the coagulation mechanism, factor Va and factor VIIIa, and thereby reduces the amount of thrombin generated; C-type lectin domain containing (575 aa)*
TEK	*Angiopoietin-1 receptor; Tyrosine-protein kinase that acts as cell-surface receptor for ANGPT1, ANGPT2 and ANGPT4 and regulates angiogenesis, endothelial cell survival, proliferation, migration, adhesion and cell spreading, reorganization of the actin cytoskeleton, but also maintenance of vascular quiescence. Has anti-inflammatory effects by preventing the leakage of proinflammatory plasma proteins and leukocytes from blood vessels. Required for normal angiogenesis and heart development during embryogenesis. Required for post- natal hematopoiesis. After birth, activates or inhibits ang [...] (1124 aa)*
PDCD1LG2	*Programmed cell death 1 ligand 2; Involved in the costimulatory signal, essential for T- cell proliferation and IFNG production in a PDCD1-independent manner. Interaction with PDCD1 inhibits T-cell proliferation by blocking cell cycle progression and cytokine production (By similarity); Belongs to the immunoglobulin superfamily. BTN/MOG family (273 aa)*
ADM	*ADM; AM and PAMP are potent hypotensive and vasodilatator agents. Numerous actions have been reported most related to the physiologic control of fluid and electrolyte homeostasis. In the kidney, am is diuretic and natriuretic, and both am and pamp inhibit aldosterone secretion by direct adrenal actions. In pituitary gland, both peptides at physiologically relevant doses inhibit basal ACTH secretion. Both peptides appear to act in brain and pituitary gland to facilitate the loss of plasma volume, actions which complement their hypotensive effects in blood vessels; Endogenous ligands (185 aa)*
SOD2	*Superoxide dismutase [Mn], mitochondrial; Destroys superoxide anion radicals which are normally produced within the cells and which are toxic to biological systems (222 aa)*
TNFRSF11A	*Tumor necrosis factor receptor superfamily member 11A; Receptor for TNFSF11/RANKL/TRANCE/OPGL; essential for RANKL-mediated osteoclastogenesis. Involved in the regulation of interactions between T-cells and dendritic cells; CD molecules (616 aa)*
OSCAR	*Osteoclast-associated immunoglobulin-like receptor; Regulator of osteoclastogenesis which plays an important bone-specific function in osteoclast differentiation; Belongs to the leukocyte receptor complex/polymeric immunogobulin receptor (PIR/LRC) family (286 aa)*

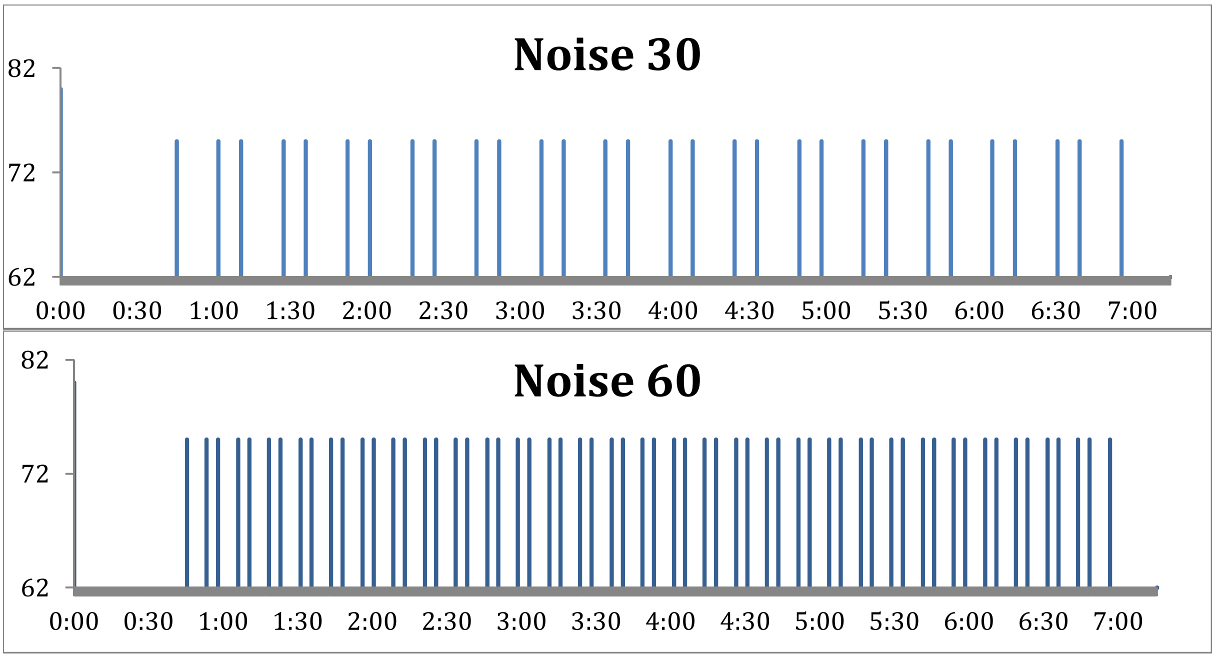

Suppl. Figure S1. Schematic representation of sound patterns. X-axis showing time in minutes, y-axis showing dB(A). Both patterns start with a calibration tone followed by 45.5 minutes of silence. Afterwards noise events occur in a long-short-long pattern. Time between events in Noise 30 approximately 15.3 minutes or 7.7 minutes, in Noise 60 approximately 6.8 minutes or 3.4 minutes. The last event is played back after roughly 416 minutes in both patterns.

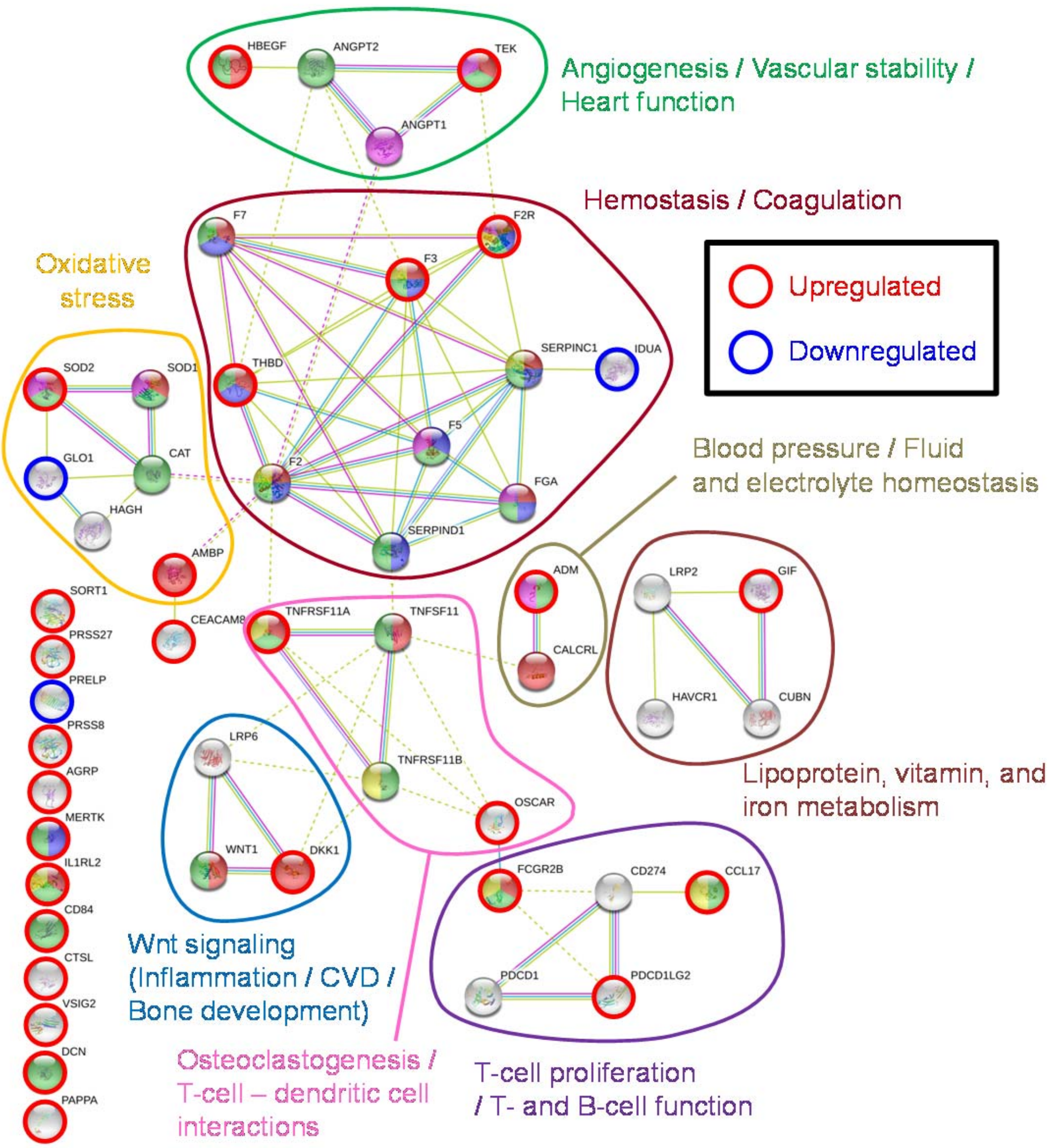

Suppl. Figure S2. Changes of the plasma proteome upon train noise exposure. STRING-database protein interaction analysis between the 31 train noise-related target plasma proteins (significantly changed as revealed by paired t-test statistical analysis). Each network node represents one protein. Blue edges (connectors) indicate protein-protein associations due to curated databases, purple edges experimentally determined associations, and yellow edges associations based on text-mining. Exposure to Noise60 caused substantial changes in the plasma proteome as revealed by cluster analysis. All measured targets are shown in **suppl. Table S2**. A targeted proteomic analysis was performed for 22 individuals showing the greatest delta between FMD in control night and FMD after Noise60. The colours within the target protein nodes have the following meaning: Regulation of response to stress (red), inflammatory response (yellow), response to stress (green), blood coagulation (blue), blood circulation (purple).

Extended Discussion on Proteomic Data

The pro-thrombotic and pro-inflammatory status characterized by the observed noise-induced protein alterations includes potential metabolism-, immune-, platelet-, and glycosaminoglycan (GAG) - mediated inflammation and changes in platelet activation. The protein with the most pronounced significant alteration, the lysosomal alpha-L-iduronidase (IDUA) participating in sequential GAG-catabolism [12], is downregulated. If regular exposure to train noise would lead to a chronic IDUA deficiency, as manifest in mucopolysaccharidosis type I, this could result in heparan - and dermatan sulfate accumulation [14] and in turn altered activities of chemokines, cytokines, and growth factors [5] and heparan sulfate proteoglycans promoted dyslipidemia and atherosclerosis [11]. Another identified protein present in lysosomes is the cysteine protease cathepsin L1 (CTSL1), which is upregulated by exposure to train noise and potentially also contributes to atherosclerosis through NLRP3 inflammasome activation [3, 6] and metabolic remodeling likely by targeting proteins involved in glycolysis, TCA cycle, and amino acid biosynthesis as shown in NIH-3T3 fibroblasts [20]. Our conclusion of a potential train-noise related pro-inflammatory and pro-thrombotic status is further strengthened by the observed upregulation of platelet-associated proteins. Besides endothelial cells, activated platelets have been shown to be a major contributor to circulating Dickkopf-related protein 1 (DKK1) levels [4, 19]. DKK1 is a quintessential antagonizing regulator of the canonical Wingless (Wnt) signaling pathway and as such a pro-inflammatory inhibitor of tissue regeneration after injury [4] as well as a mediator of the inflammatory interaction between platelets and endothelial cells promoting atherogenesis [18]. DKK1 has also been shown to promote hematopoietic stem and progenitor cell regeneration in part by decreasing the level of mitochondrial oxygen species [13]. Moreover, tyrosine-protein kinase Mer (MERTK) has been shown to be important in growth arrest-specific protein 6 (GAS6) dependent activation of platelet aggregation [2] and platelet activation is amplified by C-C motif chemokine 17 / thymus and activation-regulated chemokine (CCL17/TARC) through the C-C chemokine receptor 4 (CCR4) in the presence of low levels of primary agonists such as ADP or thrombin [8, 9, 21]. As ligand for CCR4, CCL17 further acts as chemotactic factor for T_h2 and T_{reg} cells but not monocytes or granulocytes, plays a role in T-cell development in thymus and in trafficking and activation of mature T-cells [21].

The antibody-pair for AMBP, a highly conserved glycoprotein exclusively synthesized in and secreted from the liver, targets its α1-microglobulin (A1M) chain, which is broadly distributed in serum, synovial fluid, cerebrospinal fluid, and various tissues after proteolytic processing of AMBP [1]. The upregulation of the heme- and radical-scavenger A1M together with superoxide dismutase 2 (SOD2), which might be present in exosomes [17], indicates elevated oxidative stress as it is expected in a pro-inflammatory status.

Interestingly, already temporal exposure to nocturnal train noise also caused an upregulation of the orexigenic neuropeptide agouti-related protein (AgRP). AgRP-expressing neurons in the hypothalamic arcuate nucleus normally detect caloric need and promote, upon stimulation, states of wakefulness and food-seeking behavior while decreasing the quantity and integrity of sleep [10]. Impaired sleep quality was indeed observed after both train noise patterns. If becoming chronic, such circadian disruption promotes adverse metabolic consequences such as decreased insulin sensitivity increasing the risk to develop type 2 diabetes mellitus and contributing to atherogenesis [7, 15, 16].

Other proteins among the 15 plasma proteins with most pronounced significant changes that were not mentioned above: lactoylglutathione lyase (GLO1) was down-regulated and is involved in the degradation of methylglyoxal preventing glucotoxicity and is involved in the regulation of TNF-induced transcriptional activity of NFκB; proheparin-binding EGF-like growth factor (HBEGF) was upregulated and is required for normal cardiac valve formation, normal heart function and promotes smooth muscle cell proliferation; ADM was upregulated and is a potent hypotensive and vasodilator agent with actions related to the physiologic control of fluid and electrolyte homeostasis; prostasin (PRSS8) was upregulated, possesses a trypsin-like cleavage specificity and stimulates epithelial sodium channel (ENaC) activity; low affinity immunoglobulin gamma Fc region receptor II-b (FCGR2B) was upregulated and is involved in a variety of effector and regulatory functions such as phagocytosis of immune complexes and modulation of antibody production by B- cells; carcinoembryonic antigen related cell adhesion molecule family (CEACAM8) was upregulated and belongs to the immunoglobulin superfamily; V-set and immunoglobulin domain containing 2 (VSIG2) was upregulated and is involved in epithelial cell morphogenesis as well as maintenance of gastrointestinal epithelium; gastric intrinsic factor (GIF) was upregulated and promotes absorption of

the essential vitamin cobalamin (Cbl) in the ileum; pappalysin-1 (PAPPA) was upregulated and is a metalloproteinase controlling the release of bound IGF.

Extended References

1. Akerstrom B, Gram M (2014) A1M, an extravascular tissue cleaning and housekeeping protein. Free Radic Biol Med 74:274-282 doi:10.1016/j.freeradbiomed.2014.06.025
2. Branchford BR, Stalker TJ, Law L, Acevedo G, Sather S, Brzezinski C, Wilson KM, Minson K, Lee-Sherick AB, Davizon-Castillo P, Ng C, Zhang W, Neeves KB, Lentz SR, Wang X, Frye SV, Shelton Earp H, 3rd, DeRyckere D, Brass LF, Graham DK, Di Paola JA (2018) The small-molecule MERTK inhibitor UNC2025 decreases platelet activation and prevents thrombosis. J Thromb Haemost 16:352-363 doi:10.1111/jth.13875
3. Campden RI, Zhang Y (2019) The role of lysosomal cysteine cathepsins in NLRP3 inflammasome activation. Arch Biochem Biophys doi:10.1016/j.abb.2019.02.015
4. Chae WJ, Bothwell ALM (2019) Dickkopf1: An immunomodulatory ligand and Wnt antagonist in pathological inflammation. Differentiation doi:10.1016/j.diff.2019.05.003
5. Collins LE, Troeberg L (2019) Heparan sulfate as a regulator of inflammation and immunity. J Leukoc Biol 105:81-92 doi:10.1002/JLB.3RU0618-246R
6. Duewell P, Kono H, Rayner KJ, Sirois CM, Vladimer G, Bauernfeind FG, Abela GS, Franchi L, Nunez G, Schnurr M, Espevik T, Lien E, Fitzgerald KA, Rock KL, Moore KJ, Wright SD, Hornung V, Latz E (2010) NLRP3 inflammasomes are required for atherogenesis and activated by cholesterol crystals. Nature 464:1357-1361 doi:10.1038/nature08938
7. Gan Y, Yang C, Tong X, Sun H, Cong Y, Yin X, Li L, Cao S, Dong X, Gong Y, Shi O, Deng J, Bi H, Lu Z (2015) Shift work and diabetes mellitus: a meta-analysis of observational studies. Occup Environ Med 72:72-78 doi:10.1136/oemed-2014-102150
8. Gear AR, Suttitanamongkol S, Viisoreanu D, Polanowska-Grabowska RK, Raha S, Camerini D (2001) Adenosine diphosphate strongly potentiates the ability of the chemokines MDC, TARC, and SDF-1 to stimulate platelet function. Blood 97:937-945 doi:10.1182/blood.v97.4.937
9. Gleissner CA, von Hundelshausen P, Ley K (2008) Platelet chemokines in vascular disease. Arterioscler Thromb Vasc Biol 28:1920-1927 doi:10.1161/ATVBAHA.108.169417
10. Goldstein N, Levine BJ, Loy KA, Duke WL, Meyerson OS, Jamnik AA, Carter ME (2018) Hypothalamic Neurons that Regulate Feeding Can Influence Sleep/Wake States Based on Homeostatic Need. Curr Biol 28:3736-3747 doi:10.1016/j.cub.2018.09.055
11. Gordts P, Esko JD (2018) The heparan sulfate proteoglycan grip on hyperlipidemia and atherosclerosis. Matrix Biol 71-72:262-282 doi:10.1016/j.matbio.2018.05.010
12. Hall CW, Liebaers I, Di Natale P, Neufeld EF (1978) Enzymic diagnosis of the genetic mucopolysaccharide storage disorders. Methods Enzymol 50:439-456 doi:10.1016/0076-6879(78)50048-7
13. Himburg HA, Doan PL, Quarmyne M, Yan X, Sasine J, Zhao L, Hancock GV, Kan J, Pohl KA, Tran E, Chao NJ, Harris JR, Chute JP (2017) Dickkopf-1 promotes hematopoietic regeneration via direct and niche-mediated mechanisms. Nat Med 23:91-99 doi:10.1038/nm.4251
14. Kakkis ED (2002) Enzyme replacement therapy for the mucopolysaccharide storage disorders. Expert Opin Investig Drugs 11:675-685 doi:10.1517/13543784.11.5.675
15. Leproult R, Holmback U, Van Cauter E (2014) Circadian misalignment augments markers of insulin resistance and inflammation, independently of sleep loss. Diabetes 63:1860-1869 doi:10.2337/db13-1546
16. McAlpine CS, Swirski FK (2016) Circadian Influence on Metabolism and Inflammation in Atherosclerosis. Circ Res 119:131-141 doi:10.1161/CIRCRESAHA.116.308034
17. Real JM, Ferreira LRP, Esteves GH, Koyama FC, Dias MVS, Bezerra-Neto JE, Cunha-Neto E, Machado FR, Salomao R, Azevedo LCP (2018) Exosomes from patients with septic shock convey miRNAs related to inflammation and cell cycle regulation: new signaling pathways in sepsis? Crit Care 22:68 doi:10.1186/s13054-018-2003-3
18. Ueland T, Otterdal K, Lekva T, Halvorsen B, Gabrielsen A, Sandberg WJ, Paulsson-Berne G, Pedersen TM, Folkersen L, Gullestad L, Oie E, Hansson GK, Aukrust P (2009) Dickkopf-1 enhances inflammatory interaction between platelets and endothelial cells and shows increased expression in atherosclerosis. Arterioscler Thromb Vasc Biol 29:1228-1234 doi:10.1161/ATVBAHA.109.189761

19. Voorzanger-Rousselot N, Goehrig D, Facon T, Clezardin P, Garnero P (2009) Platelet is a major contributor to circulating levels of Dickkopf-1: clinical implications in patients with multiple myeloma. Br J Haematol 145:264-266 doi:10.1111/j.1365-2141.2009.07587.x
20. Weiss-Sadan T, Itzhak G, Kaschani F, Yu Z, Mahameed M, Anaki A, Ben-Nun Y, Merquiol E, Tirosh B, Kessler B, Kaiser M, Blum G (2019) Cathepsin L Regulates Metabolic Networks Controlling Rapid Cell Growth and Proliferation. Mol Cell Proteomics 18:1330-1344 doi:10.1074/mcp.RA119.001392
21. Yoshie O, Matsushima K (2015) CCR4 and its ligands: from bench to bedside. Int Immunol 27:11-20 doi:10.1093/intimm/dxu079
